# Swept-source OCT for corneal graft quantitative evaluation in the eye bank and the correlation of the measurements to pre-excision values

**DOI:** 10.1038/s41598-022-19225-1

**Published:** 2022-09-01

**Authors:** Bartlomiej J. Kaluzny, Patryk Mlyniuk, Slawomir Liberski, Alfonso Jimenez-Villar, Anna Machalińska, Ireneusz Grulkowski

**Affiliations:** 1 Division of Ophthalmology and Optometry, Department of Ophthalmology, Collegium Medicum, Nicolaus Copernicus University, Ul. K. Ujejskiego 75, 85-168 Bydgoszcz, Poland; 2 Oftalmika Eye Hospital, Bydgoszcz, Poland; 3 Department of Ophthalmology, Poznan University of Medical Sciences, Ul. A. Szamarzewskiego 84, 61-848 Poznań, Poland; 4 Institute of Physics, Faculty of Physics, Astronomy and Informatics, Nicolaus Copernicus University, Ul. Grudziądzka 5, 87-100 Toruń, Poland; 5 First Department of Ophthalmology, Pomeranian Medical University, Al. Powstańców Wielkopolskich 72, 70-111 Szczecin, Poland

**Keywords:** Medical research, Translational research, Corneal diseases, Refractive errors, Vision disorders

## Abstract

Quantitative evaluation of the human corneal grafts stored in the tissue banks is usually limited to endothelial cell density and central thickness. Swept-source OCT (SS-OCT) is capable of measuring the central curvatures of the corneal tissue prepared for transplantation without loss of sterileness, providing insights on its refractive state. The aim of the paper is to compare in vitro SS-OCT measurements with pre-excision values. Hand-held keratometry and ultrasound pachymetry was performed on 22 corneas before excision of corneoscleral button and insertion in the vial with Eusol-C solution (AlchimiaS.r.l, Nicolò, Italy). After 12 to 36 h of hypothermic storage the corneas were examined within the vials with custom built SS-OCT system maintaining a sterile environment. The anterior and posterior central curvatures, and central corneal thickness (CCT) were measured. Rotation of the corneoscleral button was controlled by making a 6-o'clock mark during excision. Mean pre-excision CCT was 626.45 ± 28.71 µm and 468.05 ± 52.96 µm when measured with SS OCT (r = 0.55; p < 0.001). Respective values for average keratometry were 7.74 ± 0.39 mm and 7.92 ± 0.57 mm (r = 0.6; p = 0.22). Although high differences were observed in corneal thickness, keratometric radius of curvature at the flat (r = 0.42; p < 0.001) and steep (r = 0.62; p = 0.014) meridian of the anterior corneal surface, as well as corneal anterior astigmatism (r = 0.3; p < 0.001), showed good correlation with pre-excision values. SS-OCT is capable of providing quantitative evaluation of the human corneal grafts in hypothermic storage. Good correlation between curvature measurements before excision and during banking in the vial indicates its clinical utility.

## Introduction

Bilateral corneal blindness is estimated to affect 10 million people worldwide and is the third cause of blindness after cataract and glaucoma^1^. A corneal transplant, known as keratoplasty, first performed over 115 years ago by Eduard Zirm^2^, is the leading form of treatment for corneal blindness in both developed and developing countries^3^.Themost common indications for corneal graft surgery are Fuchs endothelial corneal dystrophy, keratoconus, and post-infectious keratopathy^1^. Nowadays, keratoplasty is the most frequently performed transplant surgery in the world with a high percentage of therapeutic success^2^. According to the report of The Eye Bank Association of America (EBAA), 51.336 corneal transplants were performed in the USA in 2019, which was the first place in international statistics^4^. For eye banks, the increasing number of corneal transplants is a challenge due to the necessity for efficient and precise examination of the stored corneas.

Currently, quantitative evaluation of the human corneal grafts stored in the tissue banks is usually limited to endothelial cell density and central thickness. Despite the usefulness of these methods in the evaluation of the corneas prepared for transplantation, only a limited subjective assessment of parameters reflecting the curvature of the corneas is usually performed, which limits the possibility of the precise calculation of the optical power of intraocular lenses (IOLs) before the triple procedures enabling simultaneous corneal transplantation and cataract surgery^2,5^. Incomplete assessment of the curvature of the transplanted corneas is one of the factors contributing to the occurrence of postoperative refractive errors. Among them, pre-operative, intra-operative, and postoperative factors are distinguished^6^. Inadequate evaluation of donor corneal topography, apart from the irregular surface of the recipient's eyeball and the difference in consistency and thickness between donor graft and recipient bed, is one of the pre-operative causes of the occurrence of postoperative refractive errors. The intra-operative causes include the transplant technique, type, symmetry positions, and the homogeneity tension of the applied sutures, as well as misalignment of the graft placement on the recipient bed resulting in a shift in both the horizontal and vertical plane. On the other hand, the two main postoperative factors influencing the formation of refractive errors are abnormalities in wound healing that may lead to its dehiscence and the graft melting^6^.

In recent years, there has been a dynamic development of optical coherence tomography (OCT) techniques. Devices based on OCT technology allow for quick and non-contact cross-sectional imaging of biological structures using the phenomenon of light interference^7^. Swept-source OCT (SS-OCT), the latest modification of this technology, which is capable of measuring the central curvatures of the corneal tissue prepared for transplantation without loss of sterileness, providing insights into its refractive state^8^. In previous studies, we showed the usefulness of a prototype custom-made SS-OCT in imaging wear-induced shape alternations of silicone hydrogel soft contact lenses^9–11^.This study aims to compare in vitro SS-OCT measurements of the corneoscleral button stored in the tissue bankwith pre-excision pachymetry and keratometry values.

## Material and methods

### Study design

This is a prospective, pre-post interventional study conducted at the Division of Ophthalmology and Optometry, Department of Ophthalmology, Collegium Medicum, Nicolaus Copernicus University in Bydgoszcz (Poland) in cooperation with the Department and Division of Ophthalmology of the Pomeranian Medical University in Szczecin (Poland). The study was carried out in accordance with the principles of the Declaration of Helsinki, Good Clinical Practice guidelines, the International Conference on Harmonization, and other applicable laws and regulations. In accordance with the Polish law, the corneoscleral buttons were collected from deceased who during their lifetime have not objected in writing to the Polish Central Register of Objections for Deceased Donation or verbally in the presence of two adult witnesses to the donation of their organs and tissues after death for transplantation purposes. Due to the non-invasive measurement of corneal parameters before collection in the dissecting room and the limitation of donor personally identifiable information included in the tissue collection protocol to gender, age, as well as the date, and cause of death, no additional consent to participate in the study was obtained from donors families. Importantly, the study did not affect the quality or sterility of the examined corneas, and all were used as intended for the corneal transplantation procedure.

The study was approved by the Ethic Committee on Clinical Investigation at the Nicolaus Copernicus University.

### Material

The research group consisted of 22 eyes, including 4 eyes of women and 18 eyes of men. The eyes of the deceased, whose cornea was placed in a tissue bank, were included in the study. Corneoscleral buttons with the diameter bigger when the diameter of the vial were excluded. The values of refractive and corneal astigmatism measured on the deceased (before excision) differed and were − 1.19 ± 1.46D and − 2.57 ± 1.81D, respectively. In turn, the average keratometry of the anterior surface of the cornea was 7.74 ± 0.39 mm. Table 1 presents the baseline characteristics of enrolled eyes.

**Table 1:** Baseline characteristics of enrolled eyes (pre-excision values); mean values (± SD).

	n = 22
Age (years)	51 (46.5–53)*
Spherical equivalent (D)	0 (+ 0.69–0.75)*
Refractive astigmatism (D)	− 1.19 (1.46)
Corneal astigmatism (D)	− 2.57 (1.81)
Keratometry, flat meridian, R1 (mm)	7.96 (0.39)
Keratometry, steep meridian, R2 (mm)	7.52 (0.43)
Keratometry, average (mm)	7.74 (0.39)
Central corneal thickness (µm)	626.45 (28.71)
Time from death to excision of the cornea (min)	50.09 (23.94)

*Median (Q1–Q3).

### Study protocol

All eyes were examined prior to excision the cornea from the deceased’s eye. Retinomax K-plus 5 (Righton, Japan) was used to automatically measure objective refraction and central keratometry. The thickness of the central cornea was measured using a Pachette 3 ultrasonic pachymeter (DGH Technology, USA). Then, the cornea was removed from the donor’s eye on average 50.09 ± 23.94 min after death. Immediately after removal the cornea was placed in a vial containing the Eusol-C solution (AlchimiaS.r.l, Nicolò, Italy), which is a synthetic media for corneal storage at 4 °C. The vial with an outer diameter of 26 mm has optically clear bottom which allows the evaluation of the cornea without need to transfer it to another container, which would compromise the sterility. After 12–36 h of banking in hypothermia, the corneas were examined with the custom-made SS-OCT device without removal from the vial. The measurement included the anterior and posterior curvature of the cornea as well as the central corneal thickness. The rotation of the corneoscleral flap in the vial during measurement using of SS-OCT was controlled by the mark placed before the tissue removal at 6 o’clock.

### Keratometry and CCT measurements on the deceased

Retinomax K-plus 5 (Righton, Japan) is a hand-held automated keratorefractometer that measures objective refraction and keratometry. Measurements are made monocularly, from a distance of 5 cm. This device enables the measurement of refraction in the range – 20 D to + 23 D for spheres and 12D for cylinders. Keratometry is measured over central cornea of chord length of 3.2 mm; the range is 5–15 mm for corneal curvatures and 12D for corneal astigmatism. Pachette 3 (DGH Technology, USA) is an ultrasound pachymeter that enables contact CCT measurement by taking A-scans. This device has a 20 MHz probe. The mean of 10 single measurements was taken as the CCT value. Due to the CCT measurements being performed in a contact manner, this procedure was applied after keratometry. The eye speculum has not been used. The palpebral fissure was opened manually avoiding pressure on the eyeball. In the case of difficulties with keratometry measurement, the eye surface was moistened with a 0.9% NaCl solution.

### Custom-made SS-OCT system

**Figure 1:**
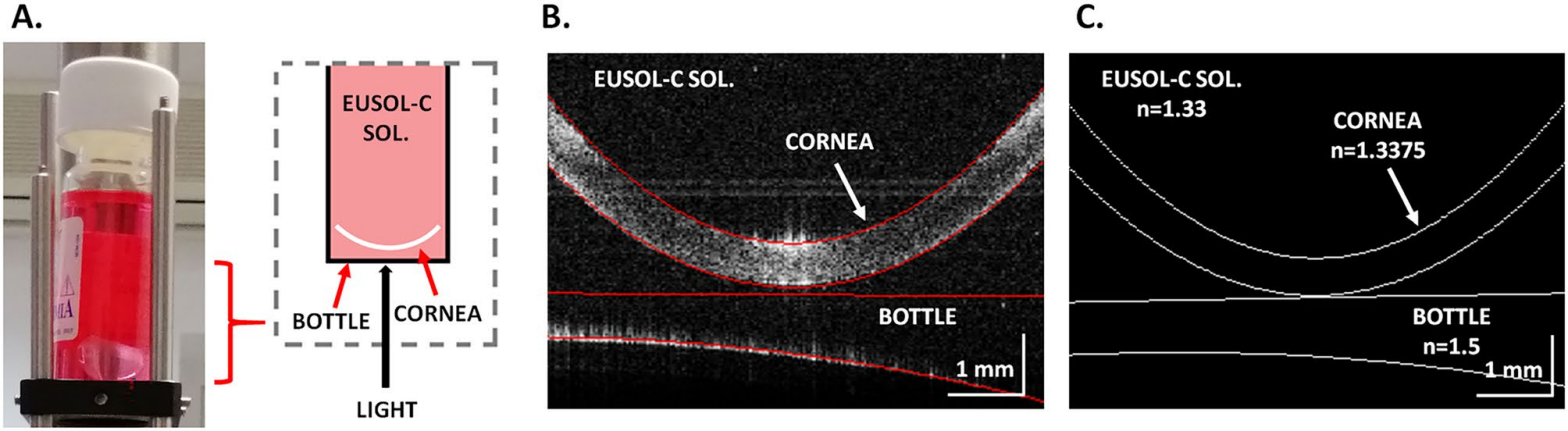
Diagram showing the method of measuring donor's corneas using a sterile vial and custom made SS-OCT device in the eye bank. (**A**) Sterile vial with the donor's cornea and its position in relation to the light source. (**B**) OCT scan obtained during experiment showing hyperreflective structure—donor's cornea—near the bottom of the vial. (**C**) Diagram with marked refractive indexes of the donor's cornea, Eusol-C solution and the vial used for calculations.

The corneas were scanned using a custom-made SS-OCT system characterized by a scanning speed of 50 kHz, and operating at a central wavelength of 1310 nm (Axsun Technologies Inc., Billerica, USA). A dual-channel acquisition board (Gage Applied Inc., Lockport, IL; Gage Compuscope 14,200, 200 MS/s, 14-bit resolution) was used to digitize interference signals. This device allowed to achieve a free-space depth (axial) range of 9.5 mm. A light beam with a power of 7.5mW was used to illuminate the tested samples, which allowed to obtain a sensitivity of 110 dB. The axial and transverse resolutions in air were 6 μm and 24.8 μm, respectively. The details of the experimental system are given elsewhere^12^. The sample arm (scanning head) of the instrument was modified to enable the measurements through the flat bottom of the glass container/vial (Fig. 1A). The three-dimensional (3D) volumetric data sets were generated using the scanprotocol that consisted of 300B-scans, each comprising a further 300 A-scans and covering 8 × 8 mm^2^ area. The OCT data were processed and meridional scans were generated from 3D data. Later on, the interfaces of the bottle and the cornea were segmented semi-automatically by fitting the curves of the 2nd order (Fig. 1B). Then, the images were corrected for light refraction.Refractive indicesof glass, Eusol-C solution and cornea wereused to calculate geometrical distances and curvatures from cross-sectional meridional images after refraction correction (Fig. 1C). Anterior and posterior curvature of the cornea was measured over central cornea using a chord length of 3.2 mm.

### Statistical analysis

The statistical analysis was performed with Statistica 13. The summary statistics for normally distributed continuous variables are presented as mean and standard deviation (SD). For variables that are non-normally distributed, the median and interquartile range (Q1, Q3) were used. The differences between continuous normally distributed variables were analysed using the t-test for independent samples. Repeated measurements variance analysis (ANOVA) was used in case of dependent samples. In case of data not normally distributed, differences were tested by the Wilcoxon test when the samples were independent or by the Friedman test if they were dependent. Pearson’s correlation coefficient (r) was used to examine the dependencies between select continuous variables. When the *P* value was less than 0.05, the results were considered to be statistically significant.

## Results

Mean endothelial cell density of the examined corneas measured in the eye bank was 2799.68 ± 327.2 cells/mm^2^. After the cornea was removed from the donor’s eye and placed in the container with Eusol-C solution, its anterior and posterior curvature at the main meridians was assessed using a custom made SS-OCT prototype. The average keratometry of the anterior and posterior surface of the cornea was 7.92 ± 0.57 mm and 6.57 ± 0.5 mm, respectively. The complete results are presented in Table 2.

**Table 2:** The keratometry of the anterior and posterior curvature of the cornea after removal from the donor’s eye measured with SS OCT in eye bank; mean values (± SD).

	n = 22
**Anterior surface of the cornea**	
Keratometry, flat meridian, R1 (mm)	8.76 (0.8)
Keratometry, steep meridian, R2 (mm)	7.08 (0.68)
Keratometry, average (mm)	7.92 (0.57)
**Posterior surface of the cornea**	
Keratometry, flat meridian, R1 (mm)	7.19 (0.88)
Keratometry, steep meridian, R2 (mm)	5.85 (0.51)
Keratometry, average (mm)	6.57 (0.5)

The keratometry values for the anterior surface of the cornea measured on the deceased were compared with the measurements of the cornea after its removal (Table 3). Higher value of the curvature in the flat meridian of the cornea was observed after its removal from the deceased’s eye (p < 0.001). In the case of the steep meridian, a slight difference in the curvature of the corneal tissue was also observed (p < 0.05). In turn, for the average keratometry, there was no significant difference between the cornea measured on the deceased and the cornea measured after excision, which were 7.74 ± 0.39 mm and 7.92 ± 0.57 mm, respectively. In addition, a significant difference in the central corneal thickness was observed (p < 0.001).

**Table 3:** Comparison of the curvature of the anterior surface of the cornea between the cornea measured on the deceased and the cornea measured after its removal from the eye; mean (± SD); r—Pearson’s correlation coefficient.

	Measurements on the deceased	Measurements of the cornea after excision	r	p
	n = 22	n = 22		
Keratometry, flat meridian, R1 (mm)	7.96 (0.39)	8.76 (0.8)	0.42	**< 0.001**
Keratometry, steep meridian, R2 (mm)	7.52 (0.43)	7.08 (0.68)	0.62	**0.014**
Keratometry average (mm)	7.74 (0.39)	7.92 (0.57)	0.6	0.22
Corneal astigmatism (D)	− 2.57 (1.81)	− 9.24 (5.36)	0.3	**< 0.001**
Central corneal thickness (µm)	626.45 (28.71)	468.05 (52.96)	0.55	**< 0.001**

Significant values are in bold.

**Figure 2:**
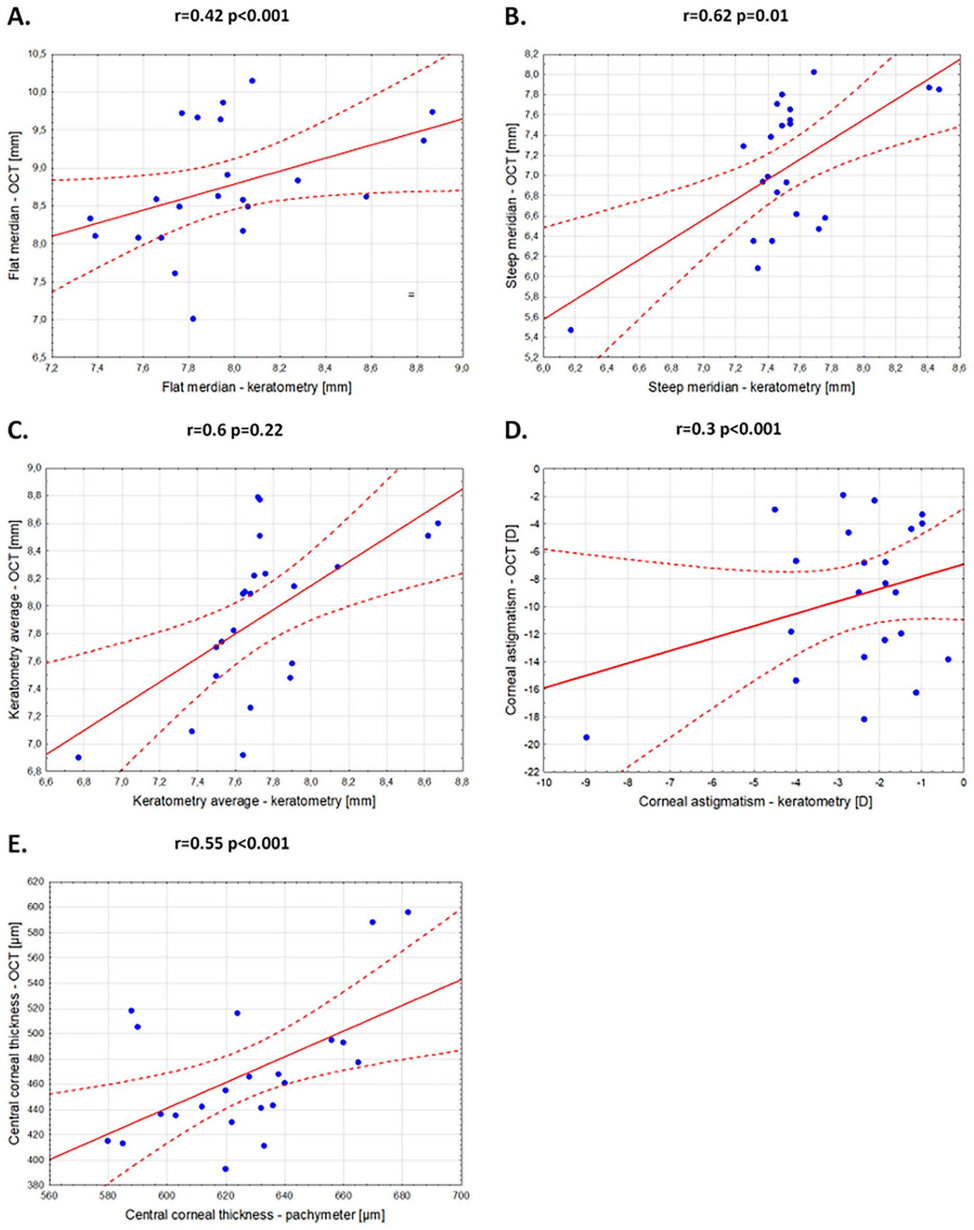
Pearson correlation plots between the anterior surface parameters of the cornea and its thickness measured on the deceased with corneal measurements made after its excision from the eye. (**A**) flat meridian; (**B**) steep meridian; (**C**) average keratometry; (**D**) corneal astigmatism; (**E**) central corneal thickness.

The correlations between the parameters of the anterior surface of the cornea and its thickness measured on the deceased with corneal measurements made after its removal from the eye was also assessed (Table 3; Fig. 2). Moderate positive correlations were observed for the flat and steep corneal meridian as well as for the central corneal thickness (p < 0.05).

## Discussion

In recent years, attention has been focused on the role of the correct qualitative and quantitative assessment of corneas prepared for graft surgery, both before removing from the donor's eye and during storage in the eye tissue bank, as an important factor influencing the prognosis of this procedure^13^. The examination of the corneas during eye banking is technically difficult due to sterility needs and therefore the desired lack of direct contact with the examined cornea^2^. The standard procedure for examining the cornea in a tissue bank, in addition to microbiological testing, includes analysis of corneal endothelial cell density (ECD) using specular microscopy, as well as visual assessment of stroma and epithelium at a slit lamp^14^. However, due to the low resolution of the image at a slit lamp and the technical difficulties related to the need to maintain sterile conditions, there is a risk of both incorrect disqualification of the cornea without significant defects, as well as qualification of the pathological corneas, e.g. with keratoconus or previously undergone refractive surgery. Moreover, the donor's medical history may not include information about any previous corneal laser surgery or corneal inflammation which may cause changes in the corneal structure and transparency^13^. Thus, the development of technology enabling the precise and repeatable evaluation of stored corneas, especially their refractive state, is critical to further progress in corneal banking and transplantation^15^.

The first device used to assess donor corneas during storage in a tissue bank was Orbscan, a topograph, based on the combining Placido-disc and slit scanning technology. However, this device only allowed to measure the anterior curvature of the examined cornea, while the CCT could not be obtained. Moreover, the examination of the cornea was impossible without its removal from the sterile container. These disadvantages made that Orbscan has been soon replaced by OCT^8^. In 2002, Neubauer et al. presented the possibility of using Spectral-Domain OCT (SD-OCT, Visante 1000 OCT, Carl Zeiss) for the evaluation of donor corneas^16^. This area of research was further developed using various modifications of OCT by Lin et al. (Time-Domain OCT, TD-OCT)^17^, Bald (SD-OCT)^18^, as well as Ghouali (Full-Field OCT, FF-OCT)^13^. In 2011, Karnowski et al. were the first to describe the application of SS-OCT in the assessment of corneal topography^19^. Janunts et al. in 2016 described the use of SS-OCT to assess the pachymetry and the anterior surface of the donors' corneas using sterile viewing chambers^8^, while Damian et al. showed the possibility of using SD-OCT for a sterile screening procedure enabling the assessment of central corneal thickness (CCT), refractive values and astigmatism of donors' corneas^2^.

In our study, we measured the corneal anterior curvature and the CCT during banking using the prototype SS-OCT to compare the values of the above parameters with the values obtained before removal from the donor's eye with the use of manual autokeratorefractometer and pachymeter. The CCT measured post-mortem was 626.45 ± 28.71 µm. This value is higher than the population norm in Caucasians reported in The Gutenberg Study, which was 557.3 ± 34.3 µm in males, and 551.6 ± 35.2 µm in females^20^. Post-mortem increase in central corneal thickness has been observed in previous human studies^21^. The most likely background of this change is dysfunction of endothelial cells and secondary corneal swelling due to the osmotic pressure gradient. Additionally, the contribution of aquaporins—proteins forming water channels, as well as endothelial and epithelial tight junctions dysfunctions was also proposed^21^. It is worth mentioning that in both animal and human studies, the initial decrease in CCT after death was observed. However, these changes resulted from the donor's eyes being open before the examination, which may cause evaporation of the tear film and could disturb the CCT measurement^21,22^.

The value of CCT measured by SS-OCT during banking was 468.05 ± 52.96 µm. The observed decrease in CCT thickness compared to the pre-excision value (p < 0.001) may be the result of the presence of dehydrating agents contained in the preservation fluid. In this study, we used Eusol-Csolution during cold storage, which contains dextran and chondroitin sulfate. Both of these compounds can raise the osmotic pressure, thereby removing excess fluid from the tissue, leading to a thinning of the stored cornea^23^.The results of previous study have shown a decrease in CCT, from 650 ± 60 µm to 590 ± 70 µm, of donor corneas, after seven days of cold storage in Optisol-GS^24^, a medium with demonstrated comparable effects on the properties of corneas to Eusol-C during short-term storage^25^. Contrary, the results of the study by Ho et al. showed an increase in CCT from 550 ± 36 µm to 591 ± 64 µm after seven days of cold storage in Optisol-GS solution^26^. In the course of the literature search, we did not find similar studies assessing changes in CCT of corneas stored in Eusol-C medium at shorter intervals, for example, 12–36 h after placement in the medium, as it was evaluated in our study. In the only available study, the morphology of the examined corneas at a slit lamp and ECD were assessed using a specular microscope^25^. Additionally, due to the different physical properties of both preservatives, especially the different osmolarity of 300 mOsm/L (range 255-345 mOsm/L) for Eusol-C^27^ and 365 mOsm/L for Optisol GS^28^, and because of the absence of a study directly comparing these preservation media, we cannot compare the dynamics of CCT changes in the stored corneas in these two solutions. Therefore further research is necessary to fully assess the dynamics of CCT changes during the initial storage period in preservation fluids.

Keratometry is the examination of the central corneal curvatures, which allows assessing its optical power. In this procedure, the length of the radius of steep and flat corneal meridians are determined^29^. Whereas, average keratometry is calculated from the mean values of radii of the steep and flat meridian^30^. Currently, measuring corneal curvatures is widely used to diagnose and monitor corneal pathologies (e.g. as astigmatism or keratoconus), during contact lens fitting, to choose the most appropriate technique for refractive surgery, as well as before cataract surgery to determine the appropriate optical power of the implanted IOL^29^. Abnormal keratometric values can lead to severe ametropia, as well as amblyopia in chlidren^29^. Thus, the efforts to achieve the best possible refractive parameters after surgery, including corneal graft surgery, are key to reaching therapeutic success. The presence of a postoperative refractive error in recipients can significantly impair vision despite a clean and healthy corneal graft. The results of the study assessing the occurrence of refractive errors after DALK and PK transplantation showed the presence of moderate to severe myopia in 25.7% and 31.4%, hyperopia in 8.6% and 17.2%, for DALK and PK, respectively^31^. Notably, after both DALK and PK moderate or severe astigmatism was found in all transplant patients^31^.

During the examination of donor corneas, we observed significant differences in both the anterior and posterior surface curvatures, as well as corneal astigmatism compared to the values of these parameters measured before trephination from the donor's eyes. The value of average keratometry measured before the excision of the corneoscleral button was 7.74 ± 0.39 mm, which corresponds to 43.5 D and is comparable to the results obtained in population studies among the Caucasian population^29^. Whereas the average keratometry of the anterior corneal surface measured in vitro by SS-OCT was 7.92 ± 0.57 mm. These values did not differ significantly, however they did not correlate with each other (Table 3). This might be caused by a relatively small range of the results obtained in our not very numerous cohort. Seventeen corneas had average keratometry within the range of 7.5 and 7.9 mm. Another potential reasons, not linked with a statistics, may could be considered. The first cause of changes in donor corneal curvatures may be the post-mortem intraocular pressure (IOP) drop which has been observed in both human and animal studies^32^. Johnson et al. showed that the human cornea is flexible and its geometry can change significantly during both an increase and a decrease in IOP^33,34^. The second reason for the observed changes is the mechanical deformation of the cornea during its removal from the donor's eye or due to positioning in the vial. Although Ousley and Terry did not show a significant effect of the trephination procedure on the change in the corneal curvatures^15^, this effect cannot be excluded and requires further research to fully evaluate this issue. The radii of steep and flat anterior meridians, as well as the value of corneal astigmatism measured before and after excision from the donor's eye were significantly different but a correlation between their changes was found (Table 3). It should be mentioned that in the case of corneas with a larger diameter, the higher values of astigmatism observed during banking may be the result of unequal pressure exerted by the walls of the glass container. Therefore, in further experiments the diameter of the vial could be larger due to the tendency to prepare bigger diameter corneoscleral buttons to facilitate their positioning on the artificial anterior chamber during layered keratoplasty. Moreover, the larger diameter of the vial may reduce the potential risk of compression of its walls on the corneoscleral button. In addition, the bottom of the container was not always perfectly flat, which made automatic segmentation difficult, while the use of an absolutely flat bottom vial could improve the segmentation accuracy of OCT scans. However, we believe that the founded dependencies indicate the possibility of using our prototype SS-OCT device in further development of the corneal transplant area, where assessing the optical power of donors' corneas before transplantation may be used in calculating the optical power of IOL in patients undergoing a classical triple procedure, as demonstrated in the study by Quintin et al.^35^. This procedure involves cataract extraction with simultaneous intraocular lens implantation and corneal transplant. It is characterized by lack of delay in visual rehabilitation, lower risk of complications, no damage to the donor corneal endothelium during re-cataract surgery, and lower costs compared to the non-simultaneous procedure in whom the calculation of the power of the implanted IOL is based on the post-keratoplasty keratometry^36^. Moreover, the use of highly advanced devices enabling the precise determination of the optical power of the transplanted corneas, and limiting the incidence of postoperative refractive errors, may reduce the incidence of graft refractive surgery, which according to the results of the study by Javadi et al. it is the most commonly performed surgical procedure in eyes that have previously undergone a corneal transplant^5^.

During the measurements on the deceased, the palpebral fissure was opened manually avoiding pressure on the eyeball. This procedure may slightly affect corneal astigmatism, especially under low IOP conditions. However, it should not affect the mean curvature of the cornea. Experiments in the pediatric population confirmed that the manual opening of the palpebral fissure and the use of wire speculum do not differ significantly in terms of the effect on keratometry^37^.

There are several directions for future research. Our findings show that our custom-made SS-OCT can be used both in theevaluation of the impact of various storage media on the graft, and can support the assessment of the impact of various trephination techniques on the changes in the keratometricparameters of the donor cornea. The use of the SS-OCT to measure corneal curvatures significantly contributes to the development of the field of corneal transplantation, along with the development of the placement methods of corneal grafts to the recipient's bed,andcan become a solution for a better donor cornea-recipient matching. It may allow significant reduction of the number postoperative complications, and importantly, significantly improve refractive results and the final visual acuity of the patients. Moreover, this technology may be use as a supplementary screening method in eye bank enabling precise examination of donor corneas during banking may avoid transplantation of not identified post-refractive cornea. It is estimated that this problem may affect up to 10% of transplanted corneas. Therefore the development of technology allowing the identification of pathological corneas with alternations that may be unidentified in currently applied screening methodsis highly desirable in this field^8^. Our study also has several limitations. As mentioned before, the technical issues related to the diameter and shape of the vial bottom could be improved in further studies. Additionally, the number of examined corneas could be higher to enhance the statistical power of the results. We also did not evaluate the epithelial status of the examined corneas. Previous studies have shown that the epithelial layer plays a role in determining the optical power of the cornea; thus, unsuspected epithelial abnormalities may affect the results^38^.

## Conclusions

The results support the conclusion that SS-OCT is capable of providing quantitative evaluation of the human corneal grafts in hypothermic storage through an optically clear bottom of the vial without loss of sterileness. Good correlation between curvature measurements before excision and during banking in the vial indicates its clinical utility. However, some changes in glass container/vial design might be considered to further improve graft qualitative evaluation in the eye bank.

## Data Availability

All relevant data generated and analyzed have been published in this article. Detailed data supporting the results can be provided by the corresponding author upon reasonable request.
